# Case report: *EML4::NTRK3* gene fusion in a patient with metastatic lung adenocarcinoma successfully treated with entrectinib

**DOI:** 10.3389/fonc.2022.1038774

**Published:** 2022-11-07

**Authors:** Chiara Lazzari, Lorenza Pecciarini, Claudio Doglioni, Federica Pedica, Ana Maria Samanes Gajate, Alessandra Bulotta, Vanesa Gregorc, Maria Giulia Cangi

**Affiliations:** ^1^ Candiolo Cancer Institute, Fondazione del Piemonte per l'Oncologia (FPO)-IRCCS, Turin, Italy; ^2^ Department of Pathology, IRCCS San Raffaele Scientific Institute, Milan, Italy; ^3^ Department of Nuclear Medicine, IRCCS San Raffaele Scientific Institute, Milan, Italy; ^4^ Department of Oncology, IRCCS San Raffaele Scientific Institute, Milan, Italy

**Keywords:** NSCLC, NTRK, EML4::NTRK3 fusion, entrectinib, NGS

## Abstract

Rearrangements involving the neurotrophin kinase (*NTRK*) genes *NTRK1*, *NTRK2* and *NTRK3* with different fusion partners have been observed in both adult and pediatric solid tumors. Larotrectinib and entrectinib have been the first tumor-agnostic compounds approved for the treatment of NTRK fusion-positive tumors. Here, we report the first case of a female patient with a diagnosis of stage IV lung adenocarcinoma harboring the *EML4::NTRK3* gene fusion, and successfully treated with entrectinib.

## Introduction

Lung cancer is the leading cause of cancer deaths worldwide (1). Non-small cell lung cancer (NSCLC) accounts for approximately 80% of cases. In the last decade, the growing knowledge of NSCLC tumor biology and the identification of targetable molecular alterations provided new tools to develop tailored medicine and improve significantly patients’ prognosis (2). Different molecular alterations, which are susceptible to targeted inhibition, have been identified in patients with NSCLC, especially in the subgroup with adenocarcinoma (2). These efforts have been translated into overall survival (OS) prolongation.

Rearrangements involving the neurotrophin kinase (*NTRK*) genes *NTRK1, NTRK2* and *NTRK3* with different fusion partners have been observed in adult and pediatric solid tumors. Larotrectinib and entrectinib have been the first tumor-agnostic compounds (i.e. drugs targeting molecular alterations regardless of the tumor histotype and localization) approved for the treatment of *NTRK* fusion-positive tumors (3). Due to the low frequency of *NTRK* rearrangements, both compounds have been evaluated in phase I and phase II basket trials.

Here, we report the first case of a female patient with a diagnosis of stage IV lung adenocarcinoma harboring the *EML4::NTRK3* gene fusion, and successfully treated with entrectinib.

## Case details

**Figure 1:**
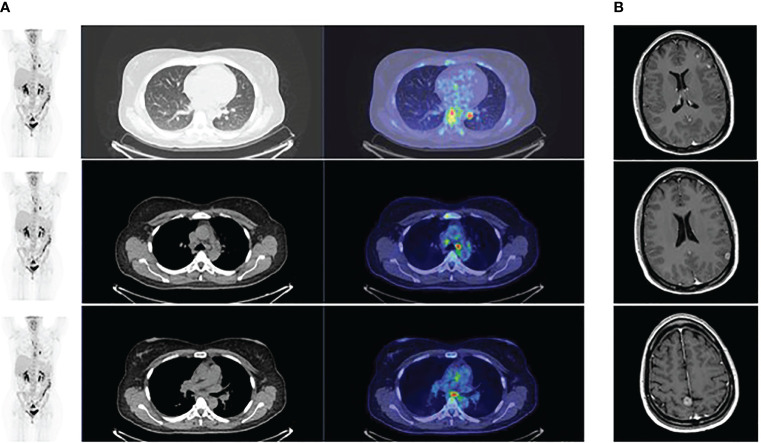
**(A)** PET scan at baseline showing the presence of lesions at the lung and the and mediastinal lymph nodes; **(B)** Magnetic brain resonance at baseline showing the presence of multiple brain metastases.

**Figure 2:**
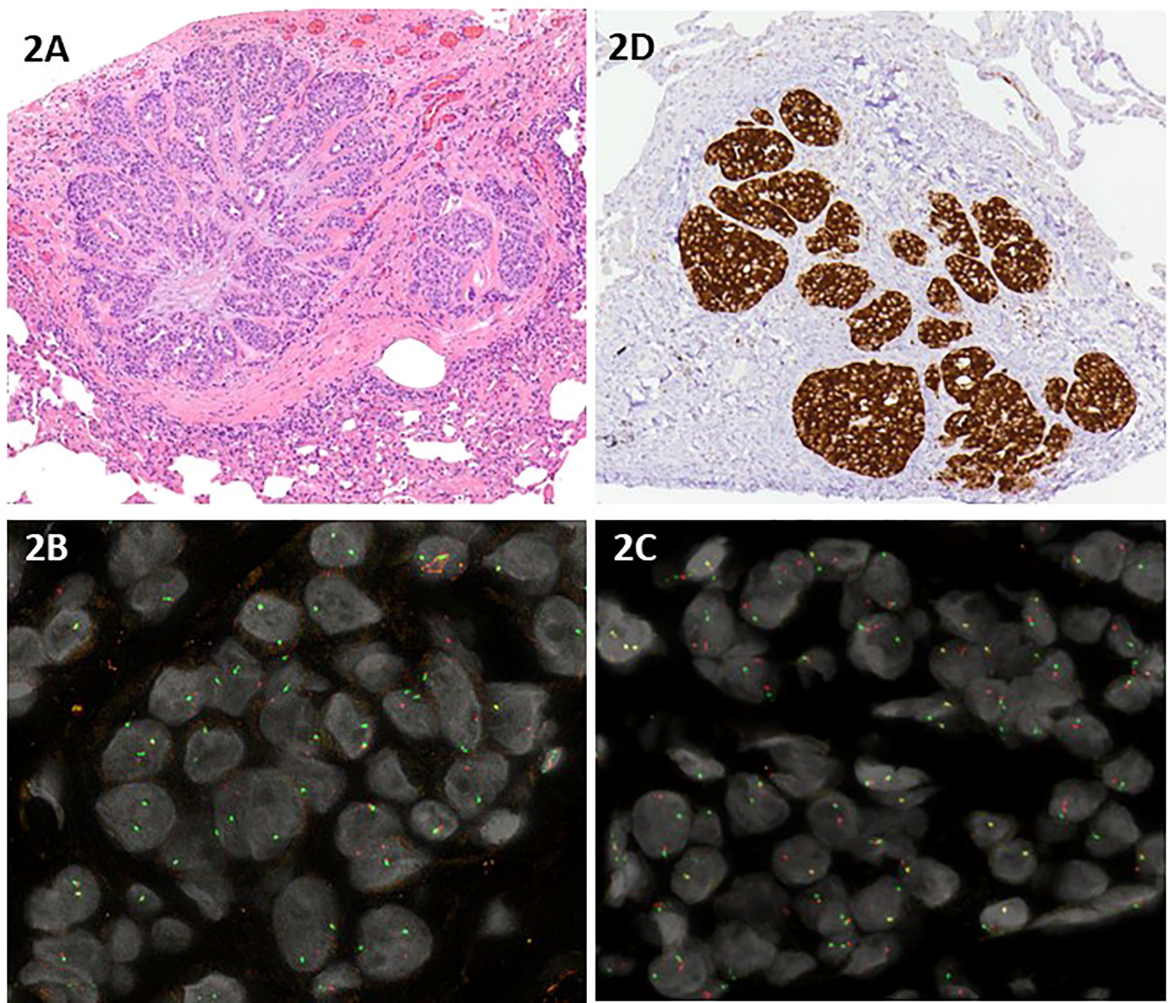
**(A)** Hematoxylin and eosin staining of acinar and solid adenocarcinoma grade 2 of the lung; **(B, C)** Interphase dual color break apart FISH using a NTRK3 probe **(B)** and a EML4 probe **(C)** displayed one normal orange/green fusion signal and one single orange signal and one single green signal in the majority of the neoplastic nuclei, indicating a rearrangement of both NTRK3 and EML4; **(D)** Immunohistochemistry staining with panTRK antibody (Roche).

A 45-year-old non-smoking female patient received a diagnosis of stage IV lung adenocarcinoma on March 2021 with pleural, bilateral lung lesions and the involvement of mediastinal lymph nodes, the sternum and the right pubic symphysis (**Figure 1A**). The magnetic resonance (MR) of the column showed the presence of metastases in the vertebral body of T9, from T6-T7 up to T9-T10, and dural enhancement anteriorly to the spinal cord of T8. The brain MR showed at least 12 lesions at sub and supratentorial sites (**Figure 1B**). To obtain a diagnosis, the patient underwent video-assisted thoracoscopic surgery with the identification of multiple alterations in the parietal, diaphragmatic, mediastinal, and visceral pleura. Histologic diagnosis described multiple localizations of acinar and solid adenocarcinoma (grade 2) of the lung (**Figure 2A**). Targeted next-generation sequencing (NGS) analysis across 161 of the most relevant genes implicated in cancer was performed using the Oncomine™ Comprehensive Assay v3 panel (Thermo Fisher Scientific) as previously described (4). The *EML4::NTRK3* gene fusion was identified (*EML4* exon2-*NTRK3* exon14), and confirmed by both fluorescence *in situ* hybridization (FISH) analysis, using ZytoLight SPEC *NTRK3* Dual Color Break Apart and ZytoLight SPEC *EML4* Dual Color Break Apart (**Figures 2B, C**, and immunohistochemistry staining with panTRK antibody (Roche) (**Figure 2D**). The same OCAv3 NGS analysis performed on matched normal tissue of the patient did not detect germline alterations.

Based on such findings, entrectinib was started.

Whole brain radiotherapy was avoided because the patient had no neurological symptoms, there was no evidence of edema in the brain MR, the number of brain lesions did not support the use of stereotactic radiosurgery and data from literature suggested the efficacy of entrectinib to cross the blood-brain barrier.

**Figure 3:**
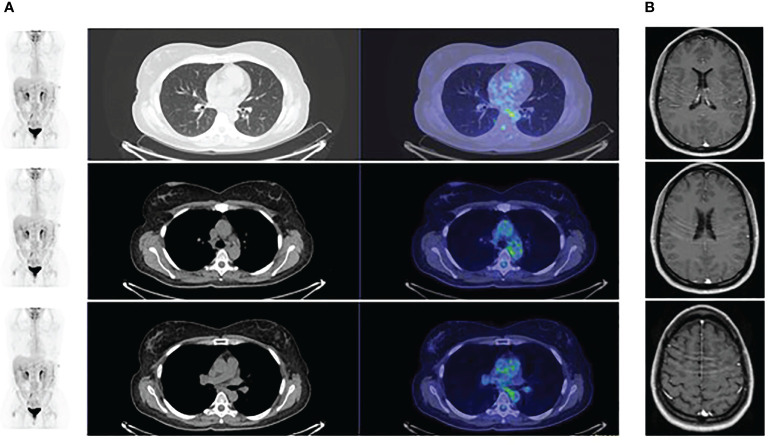
PET scan showing a partial response following 2 months of therapy.

**Figure 4:**
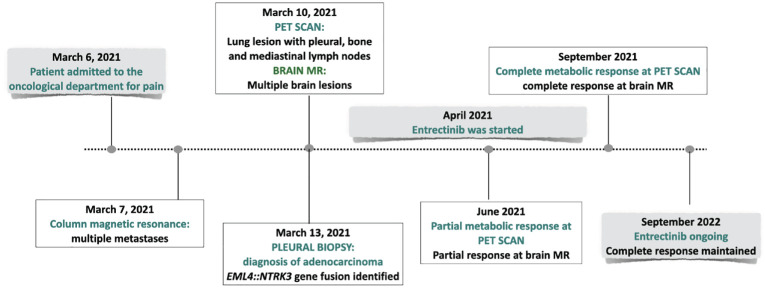
Timeline showing the clinical history of the patient.

The PET scan (**Figure 3**) and the brain MR performed two months following the beginning of treatment showed a partial response. Treatment is currently ongoing. The brain MR and the PET scan performed recently documented a complete radiologic regression. In **Figure 4**, a summary of the patient’s clinical history.

## Discussion

*NTRK* gene rearrangements are observed in different types of solid malignancies. They are the result of intrachromosomal or interchromosomal rearrangements between the 3’ portion of *NTRK1*, *2*, *3*, which includes the tyrosine kinase domain, and the 5’ portion of multiple different genes. *NTRK* rearrangements are considered agnostic tumor markers, observed in less than 1% of patients with solid malignancies. They are identified at a high frequency in rare tumors, including congenital infantile fibrosarcoma, congenital mesoblastic nephroma, secretory breast carcinoma, and mammary analog secretory carcinoma the salivary gland, and at very low frequency, in more common cancers, including NSCLC, melanoma, thyroid, esophagus, gastric or colorectal tumors (5, 6).

Retrospective data from 295676 adult and pediatric patients identified *NTRK* rearrangements in 45 tumor types and 134 different histologies (7). At least 88 rearrangements with different fusion partners have been described, being *ETV6::NTRK3* the most common, accounting for 60% of the cases (6). In NSCLC, *NTRK* rearrangements are found in less than 1% of patients, mainly in those with adenocarcinoma, but also in squamous carcinoma and large cell neuroendocrine tumors, with a variable smoking history. Here we describe for the first time the identification and characterization of an *EML4::NTRK3* fusion in a patient with lung adenocarcinoma. *NTRK3* fusions are highly enriched in patients with congenital mesoblastic nephroma, congenital fibrosarcoma, and secretory breast carcinoma. The fusion *EML4::NTRK3* has been described in two cases of infantile fibrosarcoma, one case of congenital mesoblastic nephroma (8), one patient with thyroid carcinoma (9) one 9-month old boy with congenital fibrosarcoma (10) one patient with uterus fibrosarcoma (11), two cases with colon cancer (12) and one patient with dermatofibrosarcoma protuberans (13). In carcinomas, *EML4::NTRK3* has been observed in two colon adenocarcinoma (12), and two thyroid carcinomas (9).

Larotrectinib and entrectinib have been the first compounds approved for the treatment of *NTRK* positive patients irrespective of the histology (14–16).

Larotrectinib is the first-in class NTRK inhibitor. Its safety and efficacy were initially explored in a phase I dose escalation study, enrolling 70 patients with metastatic solid tumors (15). The presence of NTRK fusions was not an inclusion criterion to enter the trial, and eight patients only carried NTRK fusions. Among these, one patient had a diagnosis of lung adenocarcinoma. Objective responses (ORR) were observed in seven out of eight patients harboring NTRK fusions and in one case with NTRK1 gene amplification. Larotrectinib was further investigated in another phase I study, including 55 NTRK fusion–positive patients. Among these, four had a diagnosis of NSCLC (17). An ORR of 75% was registered. A pooled analysis included data from three phase I/II clinical trials, enrolling 159 patients with solid tumors and NTRK fusions (15). Twelve of these had a diagnosis of NSCLC, and 13 had brain lesions. Six of the patients with NSCLC had brain metastases. Twenty-two percent of the patients enrolled were naive from previous therapy, 30% had received at least one line of chemotherapy, and 47% at least two lines of previous treatments. An ORR of 79% was observed. Among the patients with brain lesions, three cases had measurable intracranial disease. Larotrectinib determined a complete response in one patient, in the other two a partial response (46% reduction in target tumor size) and a stable disease (14% reduction in target tumor size). Similarly to larotrectinib, the more recent FDA/EMA approved entrectinib was investigated in phase I and II studies. Entrectinib inhibits *TRKA, TRKB, TRKC, ROS1* and *ALK*, and was specifically designed to cross the blood brain barrier. Its safety has been initially evaluated in two phase I trials, the ALKA-372 -001 and the STARTK-1, which showed the good tolerability of the drug. Among the patients enrolled, four carried *NTRK*-fusions. Based on the dramatic radiologic response observed in this subgroup of patients, the phase II basket STARTRK-2 study was designed to include a cohort of patients harboring *NTRK*-fusions. A pooled analysis, from the ALKA-372–001, the STARTRK-1, and the STARTRK-2 trials, enrolling 54 patients with metastatic solid tumors carrying *NTRK* fusions was subsequently published (18). Co-primary endpoints were ORR, intracranial ORR and the duration of response. Secondary endpoints included progression free survival (PFS), overall survival (OS), clinical benefit rate, time to central nervous system progression and safety. Among the patients enrolled, ten had a diagnosis of NSCLC. Thirty-seven percent were naive from previous treatment, 20% had received one line of therapy, 42% more than two lines. An ORR rate of 57% with a median duration of response of 10.4 months and a median PFS of 11.2 months were registered. In patients with NSCLC, an ORR of 70% was observed. An updated analysis, including 121 patients with 14 tumor histologies, was recently published (19). Among these, 40% had received ≥2 prior lines of therapy, 30% were naive from previous treatments, 20% had brain lesions, and 18% had a diagnosis of NSCLC. An ORR of 81% and 61% was evidenced in naive and pre-treated patients, respectively. A comparable percentage of ORR was observed in patients with *NTRK1* and *NTRK3* gene fusions (54.2% and 70.2%, respectively), while tumor reduction was shown only in one patient carrying *NTRK2* fusion. A median duration of response of 20 months was observed, with a median PFS of 13.8 months and a median OS of 33.8 months. In the subgroup of 22 patients with NSCLC, an ORR of 63.6, with a median duration of response of 19.9 months and a median PFS of 4.9 months was evidenced. Treatment was well tolerated. The majority of the adverse events registered were of grade 1, the most common being dysgeusia, diarrhea, fatigue and weight increase. Among grade≥3 adverse events, weight gain (8.3%), anemia (5.2%) and fatigue (4.7%) were those more frequently observed. Serious adverse events were reported in 12.4% of the patients, including dizziness and cognitive disorder. Five deaths, potentially related to entrectinib, were registered. All the events were of cardiovascular nature. In approximately 20% of the patients, a dose reduction occurred.

Data from *in vitro* studies comparing entrectinib to larotrectinib showed that entrectinib is a weak P-gp substrate, thus suggesting its increased efficacy in terms of central nervous system exposure (20). Entrectinib determined an intracranial ORR of 63.6% with a median intracranial duration of response of 22.1 months and a median intracranial PFS of 19.9 months in patients with measurable brain lesions included in the pooled analysis (19). The median time to central nervous system progression was 30.2 months.

These findings have opened new questions, whether withholding brain radiation in patients with brain lesions carrying targetable alterations amenable to inhibition with agents that penetrate the blood-brain barrier and result in significant control of brain disease. This represents a significant challenge, especially considering that whole brain radiotherapy might decrease cognitive function and reduce memory in patients who have a high probability of extended survival despite the diagnosis of brain metastases. Furthermore, the use of more effective therapies and the extended survival we have observed in patients with NSCLC thanks to the introduction of molecularly targeted therapies may increase the probability of CNS metastases, thus highlighting the need to design compounds able to cross the blood brain barrier and reduce the risk of brain progression. The deeper understanding of tumor biology, the introduction of next generation sequencing as a tool to molecularly classify patients, and the up-coming of next generation targeted agents, designed to penetrate the blood brain barrier, prevent brain progression, and better control disease at brain sites, will impact on the therapeutic strategy of patients with stage IV NSCLC. Patients with unstable brain lesions should be admitted to clinical trials exploring the efficacy of next generation drugs, even if not treated with radiation.

The presented case highlights the importance of obtaining a comprehensive characterization of each neoplasia, investigating also uncommon biomarkers, such as *NTRK* genes, especially in non-smokers and young (<50yrs) NSCLC patients, as they can provide useful targets for personalized therapy. Therefore, it is mandatory to utilize a reliable diagnostic methodology with high sensitivity and specificity (21, 22). Given the key therapeutical implication of *NTRK* rearrangements, it is crucial to use the best approach for the detection of tumors harboring *NTRK1/2/3* fusions. The most recognized techniques for *NTRK* fusion gene detection are immunohistochemistry, FISH, RT-PCR, and both RNA-based and DNA-based NGS. Importantly, the assay choice should take into account the type of tumor, the laboratory resources and clinical context: in tumors where *NTRK* fusions are highly recurrent, the ESMO recommendations (23) suggest the use of FISH, RT-PCR or RNA NGS panels as confirmatory techniques, whereas in testing an unselected population where *NTRK1/2/3* fusions are uncommon, either front-line NGS sequencing, when available, or screening by immunohistochemistry followed by sequencing of positive cases should be performed.

Despite formalin-fixed paraffin-embedded (FFPE) specimens being routinely used in clinical practice for both morphological evaluation and molecular characterization, it is well known that formalin fixation negatively affects nucleic acids quality by inducing fragmentation and sequence artifacts that may impact the downstream assay performance and therefore the possibility to obtain useful molecular data (24). Particular attention should be paid to obtaining adequate tissue samples and to applying standard protocols of fixation and tissue histologic processing (25), especially when RNA analysis for gene fusion detection is requested (26).

Nevertheless, tumor molecular characterization may be challenging because of the lack of adequate tumor cellularity in the tissue sample. To overcome this issue, it is necessary to implement in clinical practice the sequencing of circulating tumor nucleic acids to detect the druggable molecular alterations (27–29)

In conclusion, the *EML4::NTRK3* fusion, a rare alteration and not fully characterized in patients with NSCLC, proved to be a key target for therapy selection. This report about the efficacy of entrectinib in our patient, who obtained a complete radiologic response, is of major relevance as the literature is still very scarce about the clinical use of this drug, and moreover as it confirms the importance of molecular classification to guide the therapeutic strategy and to prolong patients’ survival.

## Data availability statement

The datasets presented in this article are not readily available because of ethical/privacy restrictions. Requests to access the datasets should be directed to the corresponding author.

## Ethics statement

The studies involving human participants were reviewed and approved by IRCCS San Raffaele Hospital, Milano (Italy). The patients/participants provided their written informed consent to participate in this study. Written informed consent was obtained from the individual(s) for the publication of any potentially identifiable images or data included in this article.

## Author contributions

VG, CL, MC, and LP contributed to the conception of the study and helped perform the analysis with constructive discussions. VG and CL contributed to manage and treat the patient. MC and LP were responsible for molecular genetic analysis and data interpretation. CD were responsible for histopathological evaluation. VG, CL, MC, and LP wrote the manuscript. All authors contributed to the article and approved the submitted version.

## Funding

This study was supported by FPRC 5X1000 2020 Ministero della Salute progetto PRO-ACTIVEMinistero della Salute Ricerca Corrente 2022.

## Conflict of interest

The authors declare that the research was conducted in the absence of any commercial or financial relationships that could be construed as a potential conflict of interest.

## Publisher’s note

All claims expressed in this article are solely those of the authors and do not necessarily represent those of their affiliated organizations, or those of the publisher, the editors and the reviewers. Any product that may be evaluated in this article, or claim that may be made by its manufacturer, is not guaranteed or endorsed by the publisher.
